# Novel Experimental Setup for Ascending Thoracic Aortic Aneurysm Inflation Testing

**DOI:** 10.3390/bioengineering13020199

**Published:** 2026-02-10

**Authors:** Hugo Mesquita Vasconcelos, Daniela Azevedo, Rodrigo Valente, Pedro J. Sousa, Tiago Domingues, Susana Dias, Rogério F. F. Lopes, Gonçalo P. Cipriano, António Tomás, Paulo J. Tavares, José Xavier, Pedro M. G. P. Moreira

**Affiliations:** 1 Institute of Science and Innovation in Mechanical Engineering and Industrial Engineering (INEGI), Campus da FEUP, R. Dr. Roberto Frias 400, 4200-465 Porto, Portugalpsousa@inegi.up.pt (P.J.S.);; 2 Unidade de Investigação e Desenvolvimento em Engenharia Mecânica e Industrial (UNIDEMI), Department of Mechanical and Industrial Engineering, NOVA School of Science and Technology, Universidade NOVA de Lisboa, 2829-516 Caparica, Portugaljmc.xavier@fct.unl.pt (J.X.); 3 Faculty of Engineering, University of Porto, 4200-465 Porto, Portugal; 4 Department of Cardiothoracic Surgery, Santa Marta Hospital, Rua de Santa Marta, 1169-024 Lisboa, Portugal; 5 Laboratório Associado de Sistemas Inteligentes(LASI), Campus de Azurém, Universidade do Minho, 4800-058 Guimarães, Portugal

**Keywords:** ascending thoracic aortic aneurysm, full-field displacement analysis, inflation testing, aortic aneurysm mechanics

## Abstract

Degraded mechanical properties in the aortic wall can lead to the formation of aortic aneurysms, potentially resulting in life-threatening ruptures. Current diagnostic criteria using maximum aortic diameter often fail to predict this critical moment, underscoring the need for more accurate patient-based prediction methods. A hospital-compatible experimental apparatus was designed for quasi-static ex vivo inflation testing of intact Ascending Thoracic Aortic Aneurysm (ATAA) specimens with 360° full-field three-dimensional digital image correlation (3D-DIC). Given hospital handling constraints, liquid pressurization was not feasible; instead, pressure was applied via a balloon-driven pneumatic system, and synchronized stereo imaging was used to measure surface displacement fields between 80 and 120 mmHg. The system was validated using a CT-derived ATAA silicone phantom. Full-field displacement measurements showed close agreement with finite element simulations, supporting the mechanical reliability of the apparatus and the repeatability of the measurement workflow. In addition, a frozen–thawed healthy porcine thoracic aorta was tested to demonstrate biological feasibility, particularly regarding the speckle application and DIC tracking, without aiming to extract tissue constitutive parameters. Overall, the setup provides a practical framework for acquiring full-field inflation-induced deformation data from intact aortic specimens in a hospital setting, enabling future studies on resected human ATAA tissue and model calibration that may contribute to more accurate methods for rupture prediction.

## 1. Introduction

In ageing developed countries, an increase in sudden unexpected deaths from heart diseases has been registered. Aneurysmal aortic rupture is a 3% cause of Japan’s sudden deaths [1]. Despite the implementation of national screening programs for early detection, 15% of diagnosed cases still result in rupture, with a fatality rate of 90% [2].

The aorta is a muscular and elastic artery known for its hyperelastic and viscoelastic characteristics. The presence of vascular smooth muscle, elastin, and collagen fibres confer its structural integrity. The distribution, organization, and percentage of elastin and collagen fibres impart highly nonlinear and anisotropic properties to the aorta.

Elastin primarily provides elasticity, allowing the aorta to stretch and return to its original shape. While the aorta itself does not contract like the heart, the smooth muscle within its walls can undergo vasomotion—subtle constriction and relaxation—to help regulate blood pressure. Collagen fibres contribute to the artery’s robustness and ability to withstand pressure [3,4,5].

The aorta, the biggest artery in the human body, can be divided into several parts. Although abdominal aneurysms are more common, ascending thoracic aortic aneurysms (ATAA) are the second most prevalent [6,7].

An aneurysm is a bulging, weakened region in a blood vessel’s wall that causes it to enlarge to more than 50% of its usual diameter. Most national health programs establish a diameter criterion for surgery, yet ruptures are known to occur before reaching this criterion, underscoring the importance of further refinement of clinical criteria [8].

Over time, numerous experimental protocols have examined the mechanical properties of aortic tissue, including aneurysmal aortas. Understanding these properties is crucial for understanding the aortic wall’s response. Changes in aortic mechanics are linked to various pathological conditions, requiring a thorough examination of tissue behaviour and structural alterations. Moreover, accurately assessing aneurysmal aortic mechanics can enhance diagnostic and treatment methodologies [4].

At present, there are three main types of aortic mechanical tests [4]. Uniaxial tests, where a sample is axially tensioned, help determine basic mechanical properties, but fail to capture the material’s anisotropy [9]. Biaxial tension tests, where the sample is tensioned in two perpendicular axes, are essential for analysing the anisotropic behaviour of aortic tissue, although they may fall short of accurately replicating the aorta’s physiological stress conditions [4]. In contrast, inflation tests offer a more comprehensive simulation of the aorta’s normal physiological conditions [10]. Inflation testing is especially useful for gaining insights into the aorta’s stress distribution and behaviour under normal operating inflation scenarios, thereby offering a closer assessment of its function within the human body [11].

Several experimental platforms have advanced ex vivo aortic pressurization and optical deformation measurement, but key limitations remain for intact ATAA testing with truly circumferential full-field kinematics. For example, Kim and Baek developed an extension–inflation apparatus with stereo vision to quantify regional deformation during pressurization, yet it was demonstrated on short proximal descending thoracic aorta segments under prescribed axial stretch (i.e., not an intact ATAA geometry with its native curvature and boundary constraints), and the measurements are not presented as a continuous 360° full-surface field [12]. Pressurization testing studies on human aortic samples (e.g., Labrosse et al.) provide valuable constitutive insights from inflation/pressurization and residual-stress assessment, but the reported outputs primarily target global quantities (stretches/opening angle/material constants) rather than full-field circumferential surface kinematics suitable for spatial validation of computational fields [13]. In ATAA-specific work, Romo et al. combined bulge-inflation with digital image stereo-correlation to obtain full-field displacements at failure; however, the protocol tests excised ATAA layers/patches rather than an intact aneurysm, thereby removing native geometric continuity and the coupled constraints that exist in the complete specimen [14]. Earlier inflation devices (e.g., Marra et al.) similarly employ bulge inflation of initially flat specimens (pressurized with water) to derive biaxial properties and rupture strength, but they do not preserve the native three-dimensional aneurysmal configuration [15]. Conversely, panoramic/multi-view DIC systems can achieve full-surface (≈360°) measurements, yet they have been demonstrated mainly for murine arteries and simplified human-scale geometries using laboratory-style optical arrangements with submerged samples/refraction correction or optical-bench implementations (e.g., Genovese et al.; Bersi et al.), rather than a compact, clinically deployable configuration designed around an intact ATAA specimen [16,17]. Taken together, the literature indicates a gap between (i) inflation tests that preserve clinically relevant ATAA geometry and constraints and (ii) optical methods that provide true 360° full-field 3D surface kinematics, motivating the present hospital-compatible system for ex vivo inflation of intact ATAA specimens with circumferential full-field 3D-DIC acquisition.

Although many inflation experimental protocols have been developed to obtain the mechanical properties of the aorta, at the time of writing, no inflation tests have been found that perform ex vivo inflation on the complete ATAA specimen. The specimen’s small cylindrical form shows a high curvature when framed and clamped for inflation testing, which poses a significant challenge because it is then very difficult to block axially, and clamping will potentially damage its border structure.

This setup is part of the AneurysmTool Project, where patients with ATAA have their aortic aneurysms surgically removed and subsequently analysed. The experimental apparatus was developed for use in a hospital environment as close to the surgical extraction as possible, which also presents operational constraints.

The goal of this manuscript is to describe the engineering design and hospital-compatible workflow of an intact-specimen inflation platform and to validate its measurement reliability. Validation is performed using (i) a CT-derived silicone phantom with finite element comparison and (ii) a healthy porcine aorta to demonstrate biological feasibility of speckle preparation and 3D-DIC acquisition. No human aneurysmal tissue is tested here, and the present results are therefore intended to demonstrate technical feasibility and measurement fidelity rather than to provide patient-specific mechanical characterization or rupture risk assessment. These clinical and biological translation steps are planned for subsequent work using surgically resected human ATAA specimens.

## 2. Experimental Setup and Methodology

**Figure 1:**
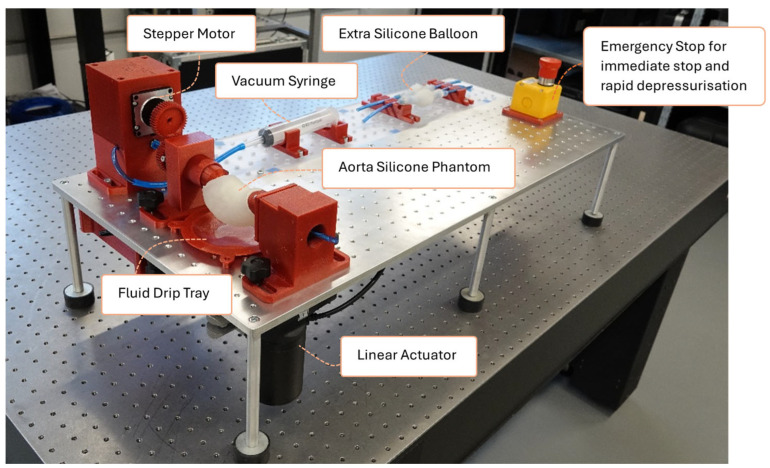
Final configuration of the experimental inflation setup for quasi-static pressurization of ATAA specimens, prepared for operation in a hospital environment. The image presents the general layout and some selected components of the system; detailed descriptions are provided in the text.

The developed apparatus applies quasi-static pressure to an ascending thoracic aortic aneurysm (ATAA) specimen and measures full-field displacements between the diastolic (80 mmHg, 10.7 kPa) and systolic (120 mmHg, 16 kPa) pressure states. The final configuration of the setup, including a silicone phantom representing an ATAA, is shown in Figure 1.

**Figure 2:**
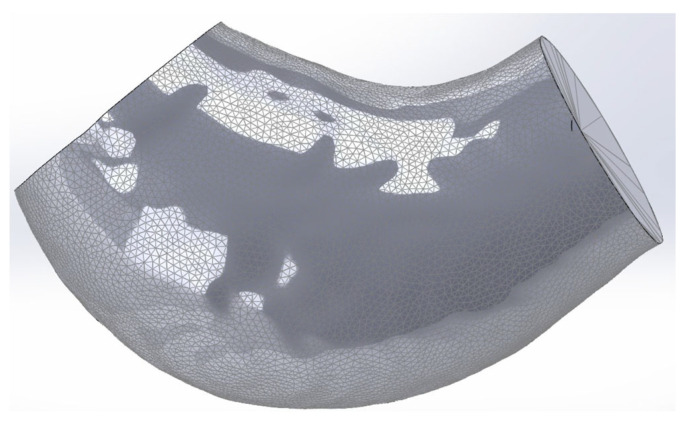
ATAA specimen geometry obtained from Valente et al. [18].

The geometry of the specimen, presented in Figure 2, was derived from an in vivo Computed Tomography (CT) scan of an aneurysmal aorta, as detailed by Valente et al. [18]. Although the obtained geometry represents a pre-stressed state, it was considered the reference undeformed geometry for this experiment.

To avoid damaging the specimen for subsequent tests, the apparatus does not impose a prescribed axial stretch or rigid end-clamping of the tissue. Nevertheless, the mounted configuration introduces practical constraints: one end is mechanically referenced by the connector/rig assembly and the pneumatic line, while the opposite end is guided by the balloon and the conical constraint rig. Additionally, the displacements measured in this setup cannot be directly compared to in vivo displacements. In vivo, the aorta is subject not only to axial constraints but also to influences from surrounding tissues and organs, which limit its expansion. These interactions, which are absent in this experimental setup, play a crucial role in the aorta’s natural deformation patterns. Consequently, the displacements observed here, reflect the mechanical response of the isolated specimen under quasi-static loading conditions and are intended for comparison with computational simulations, not direct physiological behaviour [19].

Because water or other liquids could not be used to pressurize the specimen in the hospital environment, pressurized air in a balloon was chosen to apply force to the aortic wall. The pressurizing system developed for this study operates according to the fundamental pressure-transmission principle. Although air was used instead of blood or a fluid with similar density, this substitution is considered not to impact the overall force exerted on the aortic wall, due to the force associated with the measured pressure. The system was developed to be used as close to the surgery as possible, and therefore it was not considered that the specimen’s biological properties would be altered due to drying, as it would not have time to. However, it is essential to note that the deformation observed outside the human body differs due to the absence of surrounding tissues [4,20].

**Figure 3:**
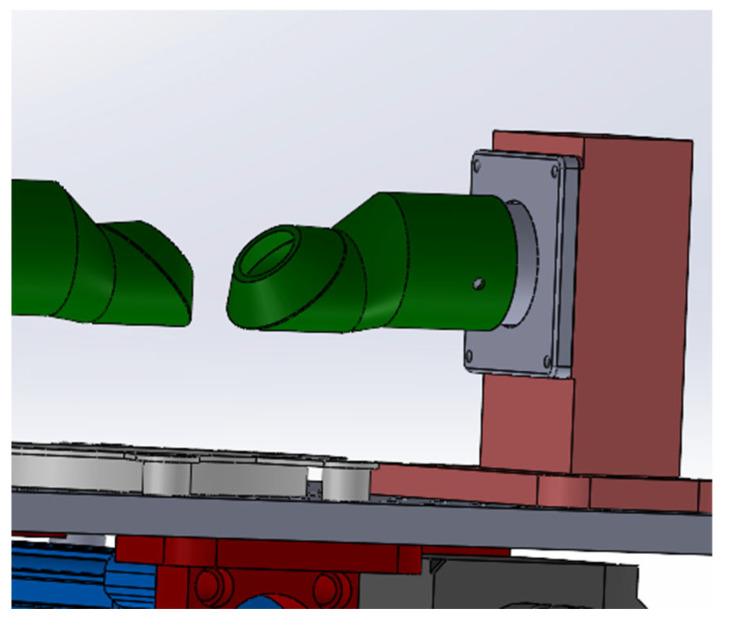
Custom-designed constraint rig used to control balloon expansion and maintain the correct positioning of the ATAA specimen during inflation, ensuring stable pressurization while allowing controlled rotational motion.

The balloon expansion enables uniform pressurization and application of force to the specimen wall. However, without constraints, the balloon would expand excessively, fail to maintain the intended pressure, and potentially rupture. To address this, two custom rigs were designed to position the balloon correctly and prevent its overexpansion. These rigs aim to closely follow the aortic curved geometry while maintaining a conical termination to accommodate specimens with diameters ranging from 20 mm to 35 mm. These rigs also allowed for controlled rotation while maintaining the restriction on balloon expansion. The design of these rigs is presented in Figure 3.

The balloon used in this setup exhibits an expansion pressure threshold below the tested pressure, at which point it begins to increase in volume. This is crucial to ensure that the internal pressure applied to the specimen comes from the imposed pressure and is not counteracted by the balloon’s structural resistance. Maintaining this distinction is vital for accurate pressure measurements. However, this setup introduces a challenge—without proper constraints, the balloon can expand beyond the specimen, preventing the desired internal pressure and leading to its rupture, as mentioned previously.

**Figure 4:**
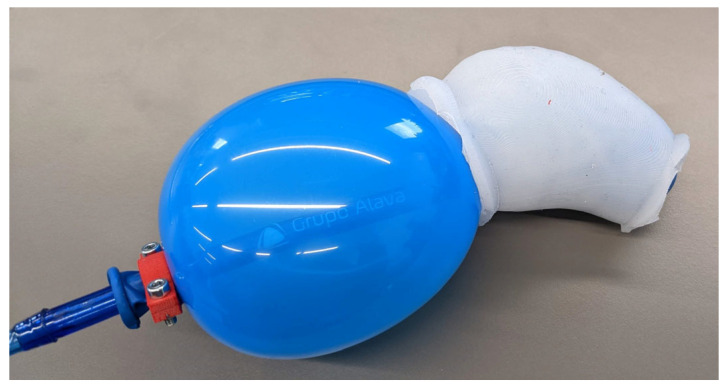
Example of uncontrolled expansion of the initial latex balloon, illustrating the inability to maintain the target internal pressure and to ensure effective pressurization of the ATAA specimen.

An example of this issue is shown in Figure 4, where an initial latex balloon expanded uncontrollably, escaping the specimen before reaching the target pressure, making it impossible to maintain the desired conditions and not allowing it to exert the intended pressure onto the specimen.

The final balloon design, developed after numerous iterations, was made from Ecoflex-00-30 silicone by Smooth-On [21]. This material was selected for its ability to withstand pressure while allowing controlled volumetric expansion, its properties are presented in Table 1. The silicone balloon exhibited a volumetric increase starting around 40 mmHg, indicating that the pressure applied to the specimen is mostly resisted by the mechanical properties of the specimen, not by the balloon’s structural integrity.

**Table 1:** Ecoflex properties [21].

**Silicone**	Ecoflex 00-30
**Mixture ratio**	1:1
**Elongation at Break**	900%
**Tear strength**	6.7 N/mm
**Specific gravity**	1.07
**Shore Hardness**	00-30

**Figure 5:**
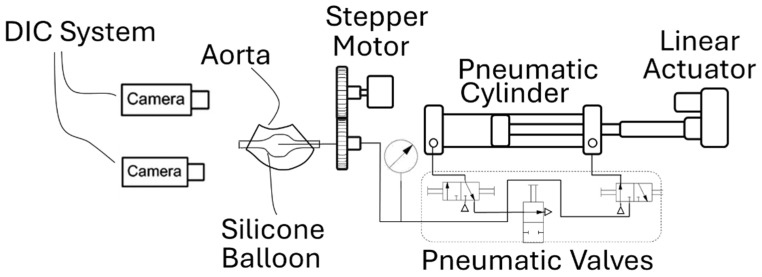
Schematic diagram of the complete experimental system, showing the pneumatic pressurization and control unit, specimen rotation mechanism, sensing components, and optical configuration for 3D-DIC measurements.

The main compressor for the system comprises a linear actuator coupled to a pneumatic cylinder. The actuator’s movement is controlled by a proportional-integral-derivative (PID) controller to maintain a constant testing pressure, and pneumatic valves provide quasi-continuous pressurization, enabling controlled, stable displacement measurements. Full-field displacement measurements are obtained using three-dimensional Digital Image Correlation (3D-DIC). A diagram of the entire setup is depicted in Figure 5.

A stepper motor rotates the specimen, enabling 360-degree measurements and ensuring complete full-field analysis. To enhance the accuracy of pressure measurements, a Honeywell SSC pressure gauge is positioned as close to the specimen as possible, as shown in Figure 5.

To measure the difference in displacement between diastolic and systolic pressures, 3D-DIC was employed. This non-contact, full-field optical technique measures 3D displacements and strains on surfaces by capturing images of the speckle-patterned specimen before and after deformation. The movement of the speckle pattern is tracked using image processing algorithms, where the speckle pattern in the deformed image is matched to the reference image [22]. The accuracy of 3D-DIC is highly dependent on the quality of the speckle pattern; therefore, an airbrush with a 0.5 mm needle was used to create a detailed, random pattern. Hansa Pro-Color water-based black and white inks approved by the hospital’s histological team were utilized to comply with biological standards [23].

Two Basler ace 2 R a2A1920-160umBAS camer (Basler, Atago Toyo Building 3F, Tokyo, Japan) as with 16 mm lenses were used to capture synchronized images. The small focal length and a larger aperture were chosen to maintain a large in-plane field of view, given the specimen’s cylindrical shape, which can easily move out of plane. Images were captured at both 80 mmHg and 120 mmHg, with measurements taken at 45° intervals around the specimen. These images were processed using Correlated Solutions’ VIC 3D (Correlated solutions, 121 Dutchman Blvd., Irmo, SC 29063, USA) [22], with a subset size of 23 pixels and a step size of 8 pixels.

To validate the developed setup, two approaches were used: evaluating the measurement principle and verifying the biological feasibility of the test.

### 2.1. Evaluation of the Measurement Principle and Accuracy

The specimen’s geometry posed a significant challenge during setup development. To address this, a CT-based model of an ascending aortic aneurysm (ATAA) from a real patient was used as the basis for the silicone phantom [18]. The phantom was created using HB Química’s Silicone HB Flex 5400, which offers properties similar to Sylgard 184 (Dow Chemical Company, Midland, MI, USA), a widely used material in the simulation of biological tissues [24].

Although Sylgard 184 is a common choice, HB Flex 5400 (HB Química, LDA - Soluções de Qualidade, 4460-886 Custóias, Matosinhos, Portugal) provided a more straightforward potting and moulding experience, facilitating proper curing and the formation of the phantom. Both HB Flex 5400 and Sylgard 184 properties are presented in Table 2.

**Table 2:** Silicone phantom materials properties.

	Sylgard 184 [25,26]	HB 5400 Flex [27]
**Elongation at break**	140%	300%
**Tear strength (N/mm)**	2.6	17.5
**Specific gravity**	1.03	1.20
**Shore hardness**	43	40

**Figure 6:**
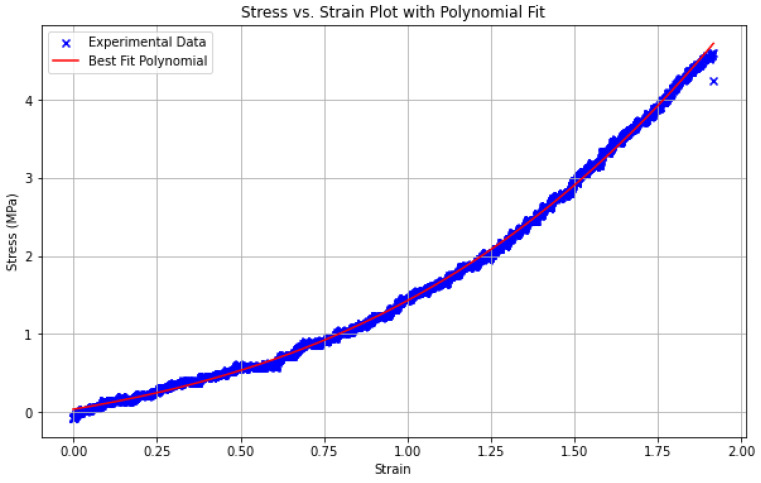
Experimental stress–strain curve of the HB Flex 5400 silicone obtained from uniaxial tensile testing. The red curve represents a polynomial fit used to highlight the overall trend of the experimental data.

To enable accurate computational analysis of the displacements, the material properties of HB 5400 silicone were empirically determined through uniaxial testing. Dogbone-shaped specimens, each 80 mm in length, were cut from the silicone phantom and tested according to *ASTM D412-16*; Standard Test Methods for Vulcanized Rubber and Thermoplastic Elastomers—Tension. ASTM International: Washington, DC, USA, 2021, with an elongation rate set to 254 mm/min [28]. The displacement data points were captured using 2D DIC in VIC and subsequently input into Abaqus’ Neo-Hookean model for analysis. The resulting experimental stress–strain response is shown in Figure 6, where a polynomial fit is included to illustrate the overall trend of the data.

The aortic structure, as previously discussed, is characterized as an incompressible, anisotropic material exhibiting hyperelastic nonlinear elasticity.

The neo-Hookean model is a hyperelastic material model that can be used for predicting the stress–strain behaviour of materials undergoing large deformations, such has silicones [29]. This model assumes non-linear elastic and isotropic behaviour, having the strain energy density equation (*W*) given by:

$$W=\frac{\mu }{2}\left(\bar{I_{1}}-3\right)+\frac{1}{d}{\left(j-1\right)}^{2}$$

where µ is the initial shear modulus, $\bar{I_{1}}$ is the first deviatoric strain invariant, *d* is the material incompressibility, and *j* is the determinant of the elastic deformation gradient [30].

Regarding the Neo-Hooke model in Abaqus, the strain energy function is represented by:

$$W=C_{10}\left(\bar{I_{1}}-3\right)+\frac{1}{D_{1}}{\left(J_{el}-1\right)}^{2}\;$$

where $C_{10}$ and $D_{1}$ are material properties, $C_{10}$ related to the shear modulus, and $D_{1}$ is related to the bulk modulus [31].

When using real uniaxial data, Abaqus performs curve fitting to match the test data to the Neo-Hookean model’s predicted stress–strain behaviour. In this fitting process, Abaqus adjusts the $C_{10}$ and $D_{1}$ parameters to minimize the error between the model’s predictions and actual test data. In Table 3 the Neo-Hooke properties obtained through Abaqus are presented for the HB Flex 5400. The Sylgard 184 and Aortic tissue properties are also presented to illustrate the resemblance of the HB Flex 5400 to them.

While the experimental setup involved inflating a balloon inside the specimen, computational simulation of this process presents considerable complexity. A direct simulation of the balloon’s expansion and the pressure it exerts on the silicone phantom requires intricate contact modelling between the balloon and the phantom material. This adds a layer of complexity compared to a simpler finite element model that applies uniform internal pressure. Despite some known differences in this method, this approach still supports a meaningful comparison with the empirical results.

**Figure 7:**
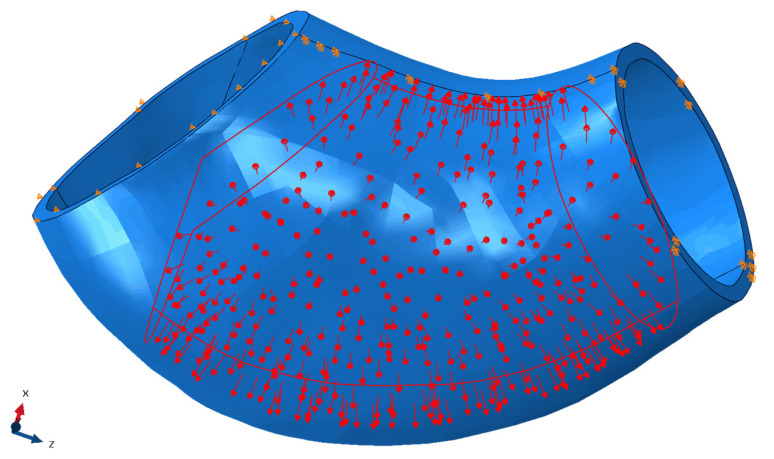
Finite element representation of the ATAA phantom illustrating the applied pressure loading and boundary conditions. The red arrows denote the pressurized region corresponding to the balloon-induced load, while the orange markers indicate the imposed constraints.

To better simulate balloon inflation, the load condition was adjusted to reflect the actual pressure distribution in the experimental setup. Rather than evenly applying pressure across the entire inner surface of the specimen, pressure was concentrated in the central region, leaving the extremities unpressurized. On the smaller side, pressure was applied up to a plane 10 mm from the surface, while on the larger side, pressure was constrained by the intersection of a plane parallel to the surface, also 10 mm away, in a plane orthogonal to the phantom’s axis. This configuration mimics the balloon’s donut-like inflation. The load condition is illustrated in Figure 7 with red arrows. An initial pressure of 80 mmHg (0.0107 MPa) was applied in the first step, followed by 120 mmHg (0.016 MPa) in the second step.

The purpose of the FE model is to reproduce the local wall deformation field under a quasi-static transmural pressure increment, rather than the global rigid-body motion that can occur in the experimental mounting. In the empirical test, the smaller-ring side remained nearly stationary relative to the camera frame (Figure 7, right side), consistent with its mechanical referencing through the connector/rig assembly and pneumatic line; therefore, this ring surface was treated as a fixed reference in the FE simulation. On the larger-ring side, experimental observations indicated that radial/normal motion was limited by the balloon–rig interaction while tangential sliding and rotation could still occur; accordingly, we restricted motion primarily along the surface-normal direction using a local coordinate system. Finally, the additional constraint on the line connecting the minimum-distance points was introduced to represent the limited lateral/axial travel imposed by the pneumatic tube/balloon system, which otherwise prevents the specimen from curving freely under load.

Since these boundary conditions caused the system to curve more than the balloon and pneumatic tube allowed, an additional constraint was applied to restrict the movement of the line connecting the points of minimum distance between the two sides. Figure 7 shows the boundary conditions with orange markers, where the smaller ring is on the right and the larger ring is on the left.

C3D10 elements were used to mesh the ascending aorta model and accurately capture its complex geometry’s mechanical behaviour. These quadratic tetrahedral elements provide higher-order interpolation of displacement and strain fields than linear elements, enabling a more detailed representation of the aorta’s intricate shape and deformation. Utilizing C3D10 elements enhances the mesh’s ability to capture finer details and localized phenomena, leading to better spatial resolution and greater simulation accuracy [35].

After performing the two-pressure simulation to obtain a proper value for comparison with the DIC analysis, the 80 mmHg computational simulation was subtracted from the 120 mmHg, and the displacement magnitude was obtained.

**Figure 8:**
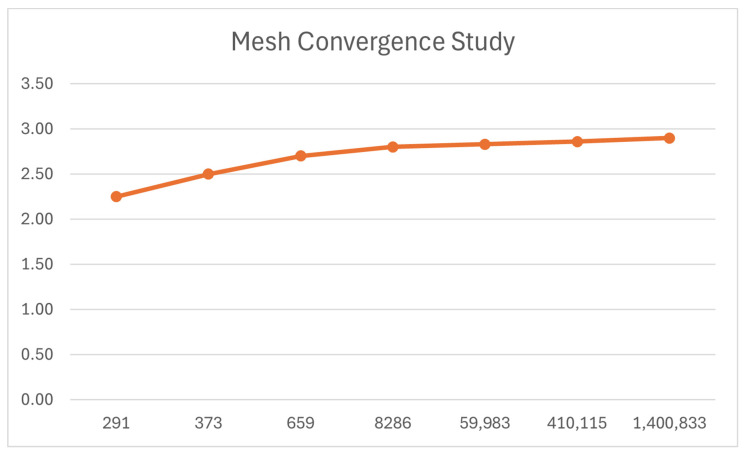
Mesh convergence study (vertical axis max displacement value, horizontal axis number of elements).

The mesh convergence study confirmed that the final mesh (1,400,833 elements) presented less than 1% variation between refinements, with consistent behaviour in both high-stress and high-strain regions. The maximum displacement was considered a practical indicator of mesh convergence, and its evolution is shown in Figure 8.

### 2.2. Evaluation of the Biological Feasibility

To assess the biological feasibility of the setup, a porcine aorta was utilized. This was a crucial step, as biological tissues present several challenges, including their high-water content, rapid ageing, stiffening, and the intricacies of applying speckle patterns for measurement.

**Figure 9:**
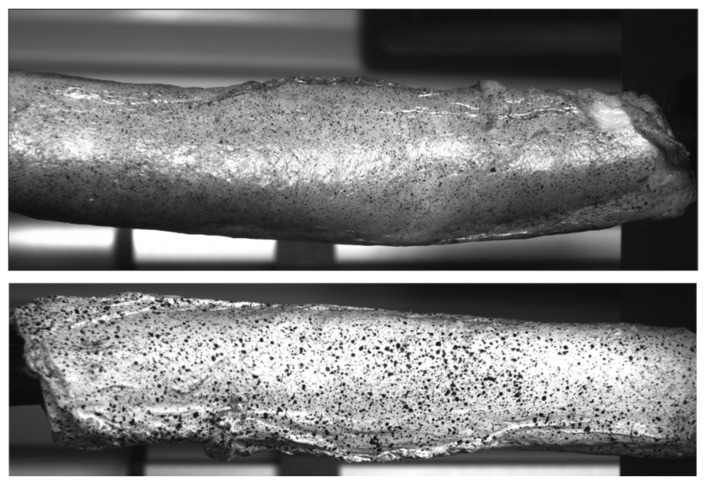
Porcine thoracic aorta: Top image without white paint, bottom image with white paint and black speckle pattern.

A porcine descending thoracic aorta, without aneurysmal deformation, was obtained from a slaughterhouse, as shown in Figure 9. Due to its anatomical characteristics, the balloon designed for the initial setup could not be used, and a new, custom balloon had to be developed specifically for this biological test.

The aorta was frozen and later thawed at room temperature. Once defrosted, it was prepared for testing by applying a white water-based base coat, followed by a black speckled pattern. Hansa Pro-Color water-based black-and-white paint was applied with an airbrush using a 0.5 mm nozzle at low pressure (0.3–1.5 bar).

These measurements were not compared to the computational analysis due to discrepancies in geometry and material properties, as the anisotropic nature of the biological tissue made direct comparison unfeasible.

Images were recorded using Point-Grey Gazelle GZL-CL-41C6M-C cameras (Gazelle, San Diego, CA, USA), equipped with 16 mm lenses (resolution: 2048 × 2048 pixels). To initialize the spatial correlation processes in digital image correlation (DIC), a stereo calibration was performed to simultaneously obtain both intrinsic and extrinsic parameters. In addition to the laboratory lighting, supplementary lighting was used to enhance image quality.

## 3. Results and Discussion

To evaluate the measurement accuracy of the setup, the first test using the silicone phantom will be presented.

The experimental setup allows the user to specify the number of images to capture per trial. In this test, eight images were taken at 45-degree intervals, first at 80 mmHg and then at 120 mmHg. The images were processed using VIC3D 9 software to analyse the total displacement between the two pressure states. Although eight images were captured, only four will be presented for clarity, followed by a comparison of the experimental and simulated results.

**Figure 10:**
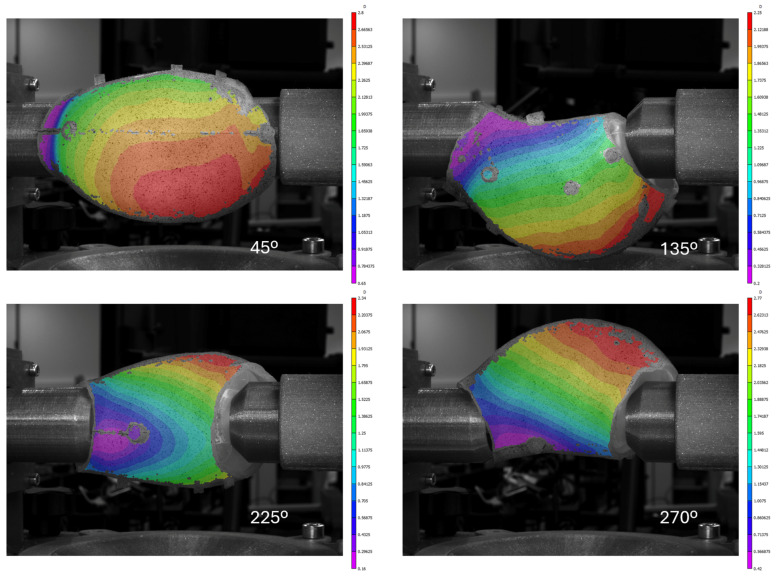
Planar colour gradient representation of the 3D magnitude total displacement.

The first set of four images displays the VIC3D analysis overlaid on a planar projection of the specimen under 120 mmHg, Figure 10. These images enable a full-field displacement analysis across the entire surface. Notably, the left side, corresponding to the smaller diameter, exhibited minimal movement. This can be attributed to the specimen’s positioning in the setup, which was slightly to the left to ensure that the balloon predominantly pressurized the larger-diameter region and did not bypass the specimen. This region was of primary interest due to its greater deformation, as confirmed through multiple trials.

From image analysis, the circumferential area along the aorta’s curved arch shows the greatest displacement, with a maximum value of 2.8 mm. A gradient in displacement is observed, decreasing towards the smaller diameter left side. In the 45-degree image, it becomes clear that the area of maximum displacement is not along the parting line but lower. This aligns with the specimen’s geometry and the production process, where the lower mould had more material, slightly offsetting the symmetry.

The presence of three sprues on the back of the specimen, visible in the images, posed challenges for VIC3D’s correlation analysis. Two of these sprues, visible in the 135-degree image, prevented proper analysis. Additionally, two sprues along the parting line are visible in the perpendicular planes of the 45-degree and 225-degree images. Despite these challenges, the VIC3D system provided comprehensive surface analysis, yielding valuable conclusions for the majority of the specimen’s surface.

Comparing the 135-degree and 270-degree images, which are nearly 180 degrees apart, the displacement patterns and gradients are consistent between the two. However, the 270-degree image exhibits a larger maximum displacement. This observation aligns with the 45-degree image, in which the side without sprues shows greater displacement. Although some variance is present in the 270-degree image, the overall values are comparable and align well with the observed trends.

While the empirical analysis provides valuable results, comparing them with computational simulations grounds the validity of the findings. In this study, the computational analysis revealed a slightly higher maximum displacement than the experimental data, with only a 1.04% difference—2.901 mm in the simulation versus 2.8 mm from the experimental results. Despite this close agreement, the locations of maximum displacement differed between the two analyses.

**Figure 11:**
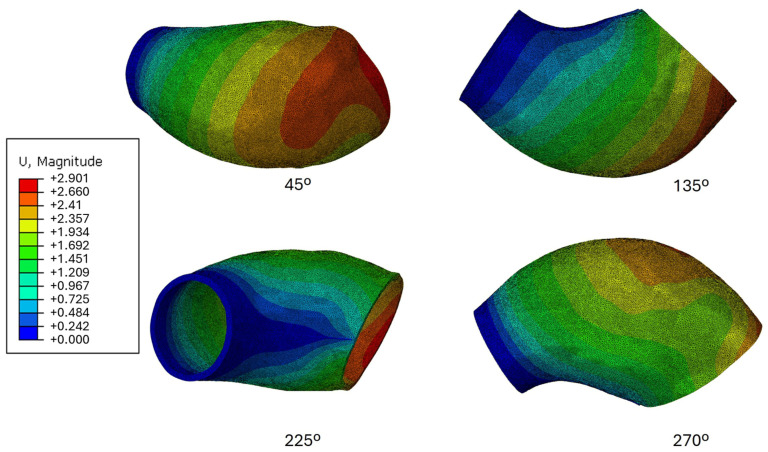
Finite element analysis results showing the total displacement magnitude of the ATAA phantom between 80 mmHg and 120 mmHg.

In the computational simulation shown in Figure 11, the maximum displacement occurred at the furthest point from the fixed, smaller arch on the right. This behaviour can be attributed to the boundary conditions, which constrained the specimen’s movement on one side, forcing expansion in the opposite direction and resulting in the highest displacement at that location.

On the other hand, the empirical analysis showed that the left side (smaller-diameter region) had the lowest movement, registering 0.6 mm, or 4% of the maximum displacement. Although initially considered negligible, this residual displacement indicates that the boundary conditions in the experimental setup were not entirely rigid. Including this small movement in the computational model would have required more complex boundary conditions, adding significant difficulty to the finite element analysis and yielding only modest improvements in the results at substantial computational cost.

The computational analysis also highlights similarities in displacement gradient patterns with the empirical results. For instance, the orientation of diagonal gradient lines in the 135° captures is notably consistent between both datasets. In the 45° capture from the simulation, a maximum displacement of 2.66 mm is observed, which is slightly higher than the empirical measurements. This discrepancy could be attributed to constraints in the boundary conditions, which may have restricted movement, or to imperfections in either the balloon or the specimen casting process. Multiple trials were conducted with varying orientations and different balloons, with no significant impact on the empirical measurements. Seven distinct balloons were developed, and the results remained consistent across trials, indicating that neither balloon orientation nor the balloon itself had a significant effect on the measurements.

However, differences introduced by the specimen pouring method could account for some of the variation between the computational and experimental results. Discrepancies between the computational model’s geometry and the real cast specimen are common, and no measurements were performed to assess the extent of the discrepancy between the silicone phantom’s poured geometry and the simulated mesh geometry. Additionally, features such as sprues, which are visible in the empirical analysis, could contribute to the observed discrepancies.

When specific areas of the specimen are compared rather than generic regions, a closer correlation in displacement magnitudes is observed between the empirical and computational results. For example, in the 135° capture, the middle section shows a displacement of 1.69 mm in the computational analysis, which closely matches the 1.609 mm measured in the empirical analysis and the corresponding gradient colours.

One of the most significant differences between the two analyses is evident in the 225° images. The computational analysis highlights the restricted movement along the boundary line between the two most proximal points of the extremities, as better illustrated in Figure 7. However, in the same orientation of the empirical analysis, some movement is visible, particularly on the right side, even though the gradient from the smaller left to the right side is similar in both analyses.

This discrepancy underscores one of the main challenges in computational analysis: without the pneumatic tube and balloon, the phantom expands unrestrictedly in directions that would not be possible in the real experiment due to these constraints. Despite this limitation, the computational analysis remains the best approximation available. Given that the actual mechanical properties of the HB5400 Flex silicone were used in the computational analysis, it is reasonable to conclude that the empirical inflation setup measurements were reliable.

A side-by-side comparison of the 0°, 90°, 180°, and 315° captures is shown in Appendix A, with corresponding images provided.

Multiple trial measurements were performed using the system with congruent results. Nonetheless, it was noted that the specimen position on top of the ballon introduced some variability.

To evaluate the biological feasibility of the setup, the second test results will now be presented.

Due to a temporary limitation of the rotation gear, it was not possible to capture the entire biological test specimen in this test, and the analysis was therefore limited to one side only. However, the displacement magnitudes obtained from this limited view correlate well with those observed in the silicone phantom test, suggesting that HB5400 Flex might be a suitable material for simulating real aortic tissue.

The porcine aorta’s geometry is significantly different from that of an aneurysmal aorta, making it unfeasible to extrapolate rotational or gradient correlations from the porcine test to the aneurysmal aorta geometry. Nevertheless, the analysis shows the characteristic donut-like pressure exerted by the balloon on the tissue. It is important to note that the balloon used in the biological test was not the same as that used in the silicone phantom experiments due to its small diameter. The porcine test required a specially developed latex balloon that covered only a small portion of the specimen, unlike the silicone phantom, where the balloon covered more than 80% of the internal surface area.

**Figure 12:**
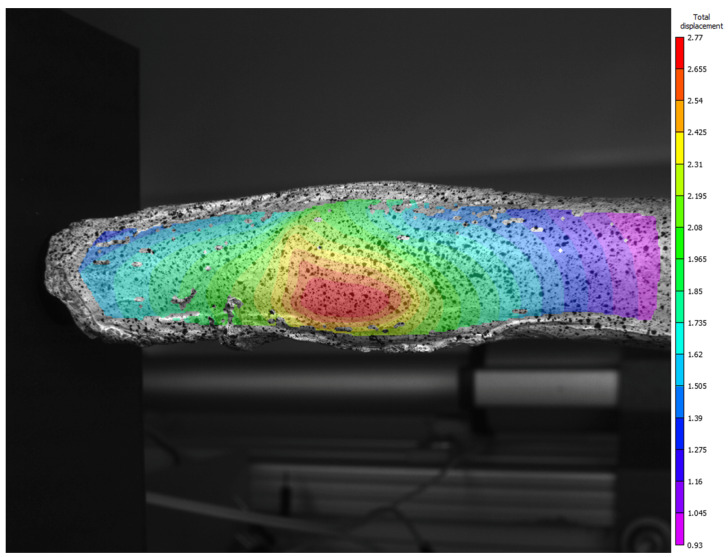
Planar colour gradient representation of the 3D magnitude of total displacement from the biological test.

The biological test demonstrated the feasibility of using paint for image correlation in a biological specimen. However, unlike the silicone phantom, which had a naturally whitish base, the porcine aorta’s pinkish hue provided insufficient contrast for the speckle pattern. As a result, it is recommended that future biological tests first apply a layer of white paint to improve contrast before adding the black speckle pattern. The full-field displacement magnitude measured during the biological inflation test is presented in Figure 12.

## 4. Conclusions

This study presents the development of an experimental setup designed to inflate an ex vivo ascending thoracic aneurysmal aorta specimen to evaluate its mechanical properties. Although in this work, no Human ATAA was tested. This work aims to present and validate this novel experimental setup. The apparatus was specifically designed to overcome the unique challenges posed by the small, curved geometry of the ATAA, which makes traditional testing methods impractical.

The use of a balloon-based air pressurization system allows simulation of physiological conditions without the need for liquid pressurization, a necessity in the hospital environment. Unlike the human body, where the aorta is constrained by surrounding tissues and organs that limit its expansion, the experimental setup lacks these factors. Therefore, the deformation patterns observed in this study do not mimic those seen in the aorta in vivo. Instead, the results provide valuable insights into the mechanical response of an isolated specimen under controlled quasi-static loading, which can be compared with computational simulations. This approach enables the study of the complete specimen properties, and although it should not be directly compared with physiological behaviour within the body, it can be used to evaluate computational models for further ATAA studies.

In this study, the Neo-Hookean material model was used to simulate the mechanical behaviour of the silicone phantom, a well-established model for silicone analysis. While the computational simulations demonstrated a close fit to the experimental data, it is important to acknowledge the inherent uncertainties in the experimental setup. These uncertainties include factors such as the precision of the material properties used, the boundary conditions, or the positioning of the specimen within the setup. Variations in specimen placement can lead to inconsistencies in applied pressure and deformation patterns, thereby affecting the accuracy of the results. Although the overall agreement between the computational predictions and empirical findings was strong, minor discrepancies highlight the need for further refinement and testing. These uncertainties suggest caution when interpreting the data, especially when considering the application of this model to biological tissues, which may exhibit more complex and less predictable behaviour than silicone.

In addition to the mechanical testing of silicone phantoms, a biological test was performed to assess the feasibility of using this setup with real tissue. The biological test supported that the system could measure aortic deformation in a clinically relevant environment. This finding supports the idea that future tests on human aneurysmal tissue can be conducted successfully using this system.

Future research will focus on using the developed setup to acquire full-field displacement measurements of surgically removed human ATAA. This will enable a more accurate assessment of the mechanical properties of ATAA tissue, thereby improving diagnostic and treatment strategies for patients with aortic aneurysms.

## Figures and Tables

**Table 3:** Phantom materials and aortic tissue properties.

	Sylgard 184 [32]	HB Flex 5400	Aortic Tissue [12,33,34]
***C*** **_10_ [N/mm^2^]**	0.3	0.3	0.05–0.3
***D*** **_1_ [mm^2^/N]**	0.5	0.41	0.45
**Poisson ratio**	0.49	0.44	0.45

**Figure A1:**
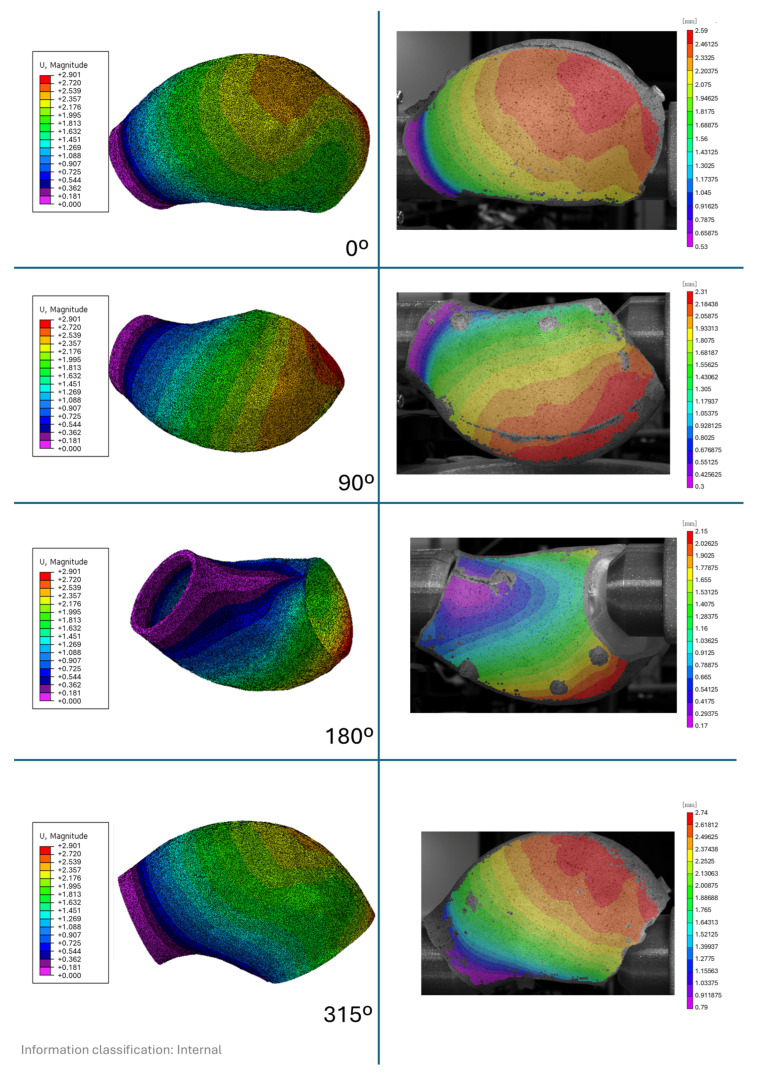
Side-by-side comparison between experimental 3D-DIC measurements and finite element (FE) simulation results for the ATAA silicone phantom at selected viewing angles (0°, 90°, 180°, and 315°). Each pair presents the planar colour map of the total displacement magnitude obtained from the pressure increment between 80 mmHg and 120 mmHg, enabling qualitative and quantitative assessment of spatial agreement between experimental and numerical displacement fields across the specimen surface.

## Data Availability

The original contributions presented in the study are included in the article, further inquiries can be directed to the corresponding author.

## Abbreviations

The following abbreviations are used in this manuscript:

ATAA	Ascending Thoracic Aortic Aneurysm
CT	Computed Tomography
DIC	Digital Image Correlation
3D-DIC	Three-Dimensional Digital Image Correlation
VIC 3D	Commercial 3D Digital Image Correlation Software (Correlated Solutions)
FE/FEM	Finite Element/Finite Element Method
ASTM	American Society for Testing and Materials
C3D10	10-node Quadratic Tetrahedral Finite Element (Abaqus element type)
